# Antimicrobial Effect of Low-Fluoride Toothpastes Containing Polyphosphate and Polyols: An In Vitro Assessment of Inhibition Zones

**DOI:** 10.3390/antibiotics12081333

**Published:** 2023-08-18

**Authors:** Igor Zen, Alberto Carlos Botazzo Delbem, Thayse Yumi Hosida, Caio Sampaio, Leonardo Antônio de Morais, Tamires Passadori Martins, Douglas Roberto Monteiro, Juliano Pelim Pessan

**Affiliations:** 1 Department of Preventive and Restorative Dentistry, School of Dentistry, São Paulo State University (UNESP), Rua José Bonifácio, 1193, Araçatuba 16015-050, SP, Brazil; igor.zen@unesp.br (I.Z.); alberto.delbem@unesp.br (A.C.B.D.); thayse.hosida@unesp.br (T.Y.H.); caio.sampaio@unesp.br (C.S.); leonardo.a.morais@unesp.br (L.A.d.M.); tamires.passadori@unesp.br (T.P.M.); douglas@unoeste.br (D.R.M.); 2 Postgraduate Program in Health Sciences, University of Western São Paulo (UNOESTE), Presidente Prudente 19050-920, SP, Brazil

**Keywords:** toothpaste, sugar alcohols, fluoride, polyphosphates

## Abstract

This study evaluated the antimicrobial effect of toothpastes containing 200 ppm fluoride (200F), xylitol (X, 16%), erythritol (E, 4%), and sodium trimetaphosphate (TMP, 0.25%), alone or in different associations, against *Streptococcus mutans* (SM), *Lactobacillus casei* (LC), *Actinomyces israelii* (AI), and *Candida albicans* (CA). Suspensions of the micro-organisms were added to a BHI Agar medium. Five wells were made on each plate to receive toothpaste suspensions at different dilutions. Toothpastes containing no actives (placebo) or 1100 ppm F (1100F) were used as negative and positive controls. Two-way ANOVA and Tukey’s HDS test were used (*p* < 0.05). For SM, the largest halo was for 200F+TMP at all dilutions, followed by the 200F+X+E toothpaste (*p* < 0.001). For LC, the overall trend showed that the polyols effectively inhibited microbial growth, and the association with the other compounds enhanced such effects (*p* < 0.001). For AI, a less-defined trend was observed. For CA, the experimental toothpaste (200F+X+E+TMP) was consistently more effective than the other treatments, followed by 200F+X+E (*p* < 0.001). The association of polyols and TMP in a low-fluoride toothpaste effectively reduced the growth of cariogenic micro-organisms (SM, CA, and LC), suggesting that this formulation could be an interesting alternative for children due to its low fluoride content.

## 1. Introduction

Dental caries is a chronic, biofilm-modulated disease, which has increasingly affected children worldwide [1], being associated with an increase in fermentable carbohydrates intake, and deficient removal or disorganization of dental biofilm. It is a sucrose-dependent biofilm disease, whose onset is related to the interaction of acidogenic and aciduric micro-organisms in the oral cavity [2].

Among the main micro-organisms involved, *Streptococcus mutans* stand out for their ability to colonize dental surfaces, metabolize carbohydrates, and produce lactic acid [3]. Other bacteria are related to the disease’s onset and progression, including *Lactobacillus* and *Actinomyces* [4]. In addition, the fungus *Candida albicans* has been reported to contribute to the formation and development of cariogenic biofilms, especially in dentine cavities, as its proteolytic enzymes are able to break down collagen molecules [5,6].

The association of mechanical and chemical methods is an effective method to reduce biofilm formation [7,8,9]. Among them, toothpastes present in their composition active agents that have mineralizing and/or antimicrobial action, such as fluoride (F) [8,9]. Furthermore, the addition of phosphate salts to F-containing toothpastes has been shown to boost the effects of F. In vitro and in situ studies have shown that low-F toothpastes containing sodium trimetaphosphate (TMP) have similar effects to those of a conventional formulation (1100 ppm F) on enamel de- and re-mineralization [10,11], and on F and calcium levels in the dental biofilm [10,12]. In addition to its promising effects on enamel, combined treatments of TMP and F, administered as solutions, have also been demonstrated to substantially affect the metabolism and matrix composition of dual-species biofilms of *C. albicans* and *S. mutans* [13], which can be mainly explained by its chelating property that exerts an influence on the permeability of the bacterial cell wall [14,15].

Besides the strategies above, products of natural origin, such as polyols, are used as sugar substitutes, which have the ability to reduce the growth and attachment of *S. mutans* to surfaces and reduce the volume of dental biofilm [16]. Literature shows a synergism between xylitol and F on the inhibition of acid production by *S. mutans*, with significantly higher effects than the actives alone [17]. Furthermore, erythritol has been demonstrated to affect even further microbial parameters compared with xylitol, reducing the expression of bacterial genes involved in sucrose metabolism and the overall number of dental caries, and serving as a suitable matrix for subgingival air-polishing to replace traditional root scaling, affecting both caries- and periodontitis-related pathogens, besides being suggested as an interesting alternative due to the lack of adverse events associated with its usage [18,19,20]. Although the mechanism of erythritol is not fully understood, the antimicrobial effects of these polyols can be considered to be by the fructose phosphotransferase system, which leads the cell to a high consumption of energy, causing it to not produce enough energy for the necessary bacterial conservation [21,22,23].

Considering the advantages of the above-mentioned strategies used alone and the need to develop safer and more effective formulations for children, this study’s objective is to evaluate in vitro the antimicrobial effect of toothpastes containing F at low concentration (200 ppm), xylitol, erythritol, and TMP, alone or in different associations on the suppression of the growth of *S. mutans, Lactobacillus casei*, *Actinomyces israelii*, and *C. albicans* strains, by assessing inhibition zones in agar culture media. The study’s null hypothesis was that the antimicrobial effects of the toothpastes associating two or more compounds would not be significantly different from the toothpastes containing the isolated actives.

## 2. Results

**Figure 1:**
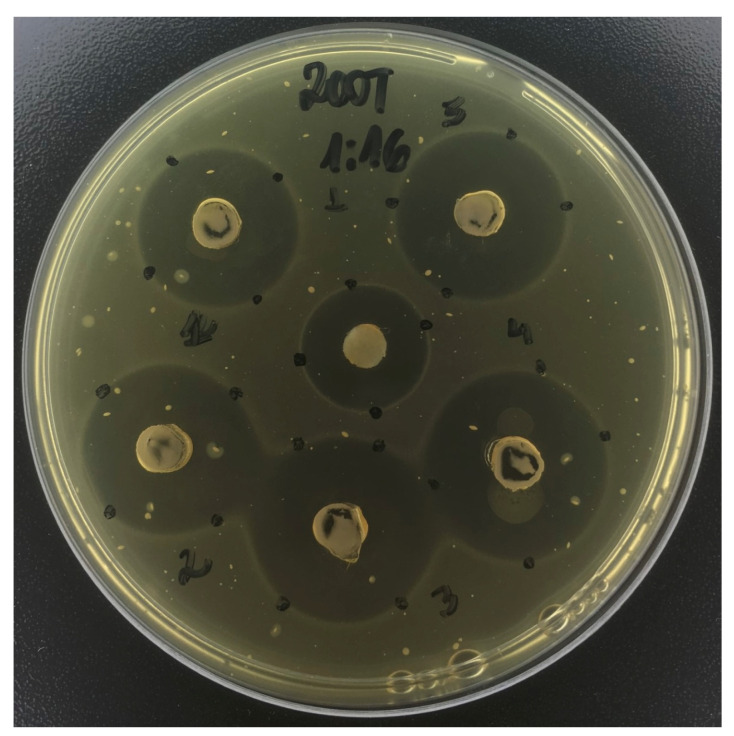
Inhibition zone promoted by the toothpaste containing 200 ppm fluoride + sodium trimetaphosphate (0.25%), at 1:16, on *Streptococcus mutans*. This figure illustrates a considerable zone of inhibition promoted by the toothpaste.

Overall, for *S. mutans,* the largest inhibition halo was observed for 200F+TMP (Figure 1) in all dilutions (*p* < 0.001). Nonetheless, the effects of the polyols used together, as well as their association with 200 ppm F promoted larger zones of inhibition compared with the other groups (*p* < 0.001), with sustained effects for all the dilutions (Table 1).

**Figure 2:**
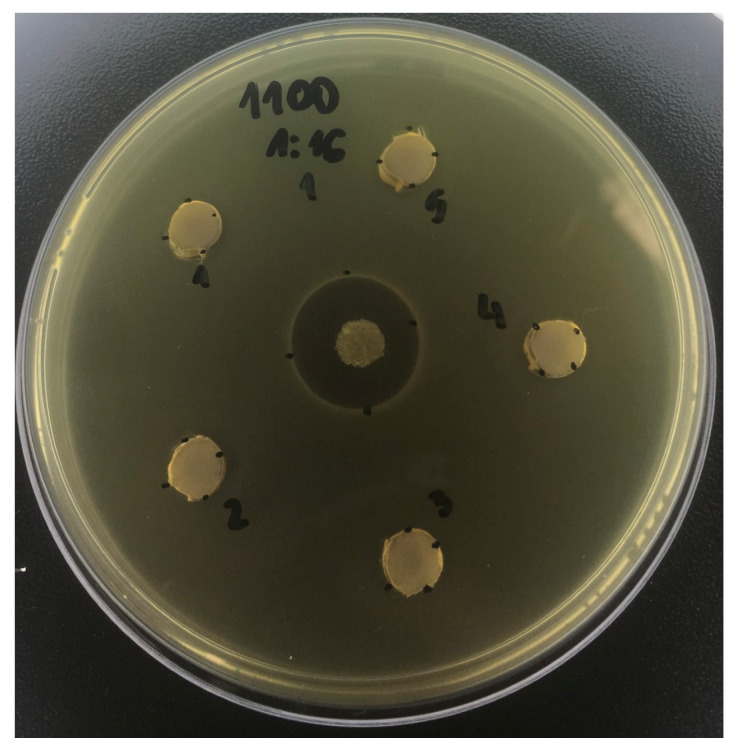
Inhibition zone promoted by the toothpaste containing 1100 ppm fluoride, at 1:16, on *Lactobacillus casei*. This figure illustrates the lack of effect of this formulation on the strain.

For *L. casei*, the general pattern was that the polyols, alone or coadministered, were effective in inhibiting microbial growth, and their association with the other actives potentiated the antimicrobial effects (Table 2; *p* < 0.001). In this sense, larger inhibition halos were observed for toothpastes containing both polyols and F combined (at most all the dilutions), both polyols and TMP combined (for the dilutions 1:2, 1:4, and 1:8), and for the experimental toothpaste (at the 1:1, 1:2, and 1:4 dilution). In addition, for most of the dilutions, 1100F did not promote a significant effect on this strain (Figure 2; *p* > 0.05).

For *A. israelii*, no defined trend was observed for treatments at the 1:1, 1:2, and 1:4 dilutions (Table 3). For the remaining dilutions (1:8 and 1:16), toothpaste containing 200 ppm F promoted the largest zones of inhibition compared with the other treatments (*p* < 0.001). In general, dentifrices containing polyols, regardless of the combinations, were not capable of producing significant inhibition zones at most of the dilutions (*p* > 0.05).

Conversely, for *C. albicans*, the experimental toothpaste (containing all actives) promoted a consistently higher inhibitory effect than the other treatments for all dilutions (except for 1:8), followed by 200F+X+E (Table 4; *p* < 0.05).

## 3. Discussion

In this study, the antimicrobial effect of toothpastes containing 200 ppm F, xylitol (16%), erythritol (4%), and TMP (0.25%), isolated or in different associations on *S. mutans*, *A. israelii*, *L. casei,* and *C. albicans*, was determined using an in vitro screening model. The results show that the toothpastes containing two or more actives promoted significantly larger inhibition zones than the isolated compounds for most of the pairwise comparisons, whose effects depended on the strain analyzed. Thus, the null hypothesis was rejected.

*S. mutans* was one of the strains chosen due to its direct involvement in dental caries onset and progression. For this strain, while toothpastes containing TMP alone or F at low concentrations promoted only modest antimicrobial effects, the largest inhibition halos were achieved by the association between 200 ppm F+TMP, for all dilutions (Table 1; Figure 1). These findings show the synergism between the two compounds, which is in agreement with CFU data of *C. albicans* and *S. mutans* [13]. Furthermore, this association was shown to reduce the total biofilm biomass and extracellular matrix components, and also promoted the highest pH values both before and after cariogenic challenges [23]. In vitro and in situ studies have also shown that the association between F and TMP leads to synergistic effects on de- and re-mineralization [10,11,23], reason by which such combination was assessed in the present study.

On the other hand, xylitol and/or erythritol promoted modest reductions in *S. mutans* growth. Literature reports that xylitol affects *S. mutans* cells by inhibiting glycolytic enzymes, which leads to a reduction of growth and acid production [24]. For erythritol, however, its mechanism is not fully known, although data have observed a superior effect for this polyol for some caries-related endpoints in comparison to xylitol [18]. Despite the fact that the reasons for the small effects of the polyols on *S. mutans* in the present study are not apparent, it is possible that the effect of these actives on this bacterium would be related to their antibiofilm action (e.g., extracellular polysaccharides production) rather than an inhibitory effect. Moreover, data have demonstrated that the sensitivity of the *S. mutans* strains to polyols varied markedly, and some strains were more sensitive to xylitol and some to erythritol, leading to the assumption that the effects of these polyols may be strain-dependent [25]. In fact, other natural or natural-like products have been demonstrated to suppress the growth of *S. mutans.* A recent in vitro study showed that olive oil mouthwashes were able to inactivate *S. mutans* in a protocol simulating salivary dilutions in the oral cavity. In light of this trend, the combination of the polyols with other promising actives such as the above-mentioned one, in addition to their incorporation to other vehicles, could be an interesting alternative for verifying their antimicrobial effects [26].

As for *L. casei*, conflicting evidence on the effects of xylitol is available from clinical studies. While some authors showed that xylitol’s continued use promoted reductions in *Lactobacillus* counts in saliva [27,28,29], others showed that the use of xylitol-containing gum [30] or toothpaste [31] did not reduce *Lactobacillus* counts compared with control groups. The different methodologies, vehicles of administration, and study duration hinder a direct comparison between studies, as these variables may have impacted the results. In the present study, the largest inhibition zone was observed for the experimental and xylitol toothpastes (at a 1:1 dilution). Such effects might have resulted from xylitol’s action on this strain following a similar mechanism to that described for *S. mutans*, in which it is related to the phosphotransferase system that inhibits the conservation of the bacteria and further growth [32,33,34,35].

**Figure 3:**
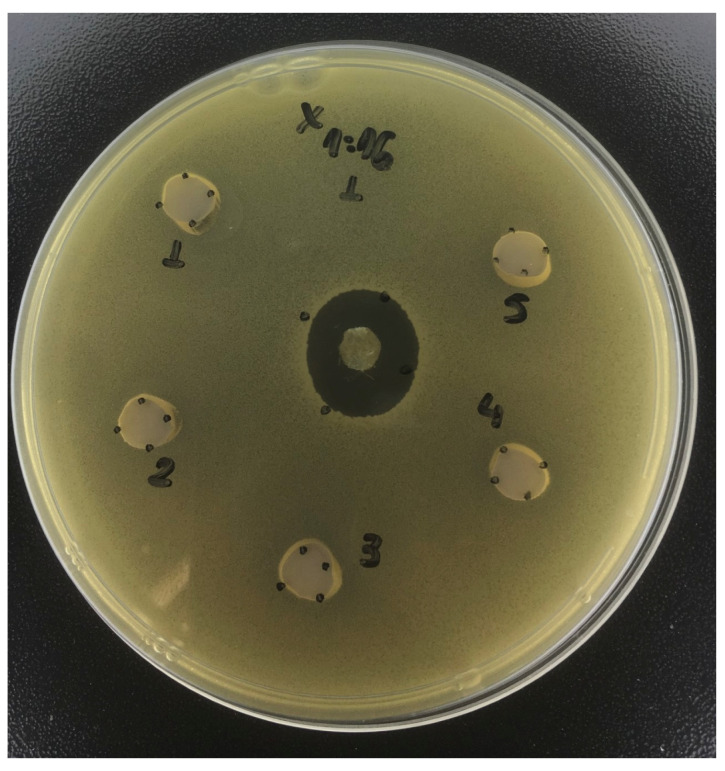
Inhibition zone promoted by the toothpaste containing xylitol, at 1:16, on *Actinomyces israelii.* This figure illustrates the lack of effect of this formulation on the strain.

Regarding *A. israelii*, no striking pattern was observed among the groups on the inhibition halos compared with the placebo toothpaste, for any of the tested dilutions (Table 3, Figure 3). This is in accordance with previous results showing that xylitol in the presence of glucose did not inhibit the growth of *Actinomyces* [36]. This fact can be explained by the low acidogenicity and acid tolerance of this strain [21]. They also reflect the status of non-cariogenic micro-organisms compared with *S. mutans* and *L. casei* [21,22,23,24,25,26,27,28,29,30,31,32,33,34,35,36,37].

**Figure 4:**
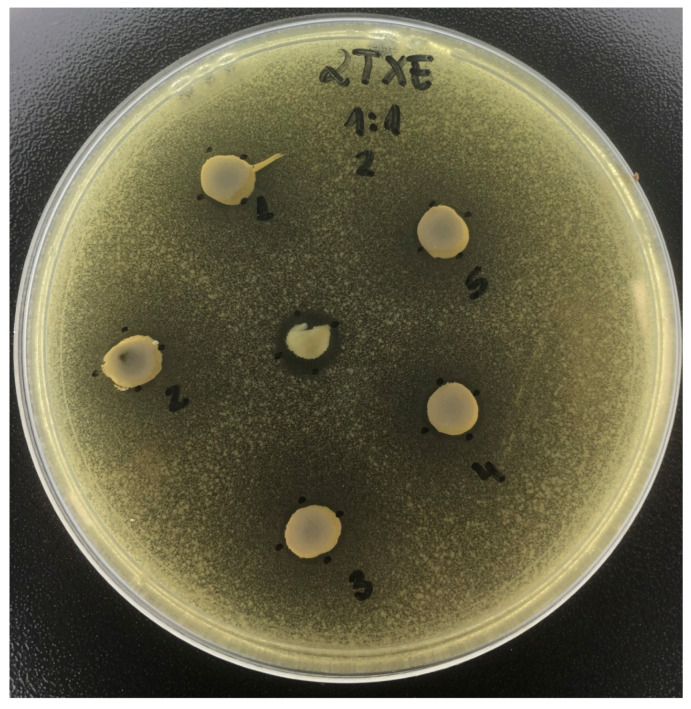
Inhibition zone promoted by the EXP (toothpaste containing 200 ppm of fluoride + xylitol (16%) + erythritol (4%) + sodium trimetaphosphate (0.25%)), at 1:1, on *Candida albicans*. This figure illustrates the substantial inhibition zone produced by this treatment.

In contrast, *C. albicans* has recently been used in in vitro biofilm protocols to reproduce conditions that better resemble those of cariogenic biofilms in vivo [13,23] as this fungus has been identified in biofilms collected from individuals presenting cavitated caries lesions [6]. In the present study, the highest inhibition halos were promoted by the experimental toothpaste (at the 1:1, 1:2, and 1:4 dilutions; Table 4; Figure 4), so that the simultaneous action of all compounds could explain such effects. Previous studies have shown that xylitol presents an inhibitory effect on *C. albicans* growth [38], which is due to an impaired nutrient absorption required to maintain yeast viability. The inability of *C. albicans* cells in catabolizing or excreting xylitol products accumulated in the cytoplasm also explains such effects, as such products lead to an increased osmotic strength and cell swelling [39,40]. Regarding erythritol, this compound was shown to increase the effect of benzethonium chloride against *C. albicans*, leading to the assumption that erythritol can help to disperse biofilms, favoring the penetration of fungicidal agents and weakening the microbial bond to surfaces [41]. However, the literature on this topic is scarce, pointing to the need for more comprehensive studies on the relationship between erythritol and this fungus.

A previous study showed that the association of F (500 ppm) and TMP on a biofilm of *S. mutans* and *C. albicans* was shown to promote alterations on the extracellular matrix and biofilm architecture compared with 1100 ppm F without TMP [13]. Such effects were attributed to the interaction of these compounds, which directly acted on the inhibition of bacterial enzymes and the reduction of intra- and extra-cellular polysaccharide production [42,43]. Based on this rationale, it could be hypothesized that the inhibitory effect for both *S. mutans* and *C. albicans* in the present study would result from the interaction (in different associations) of xylitol, erythritol, TMP, and/or F, through different mechanisms. Previous studies reported that these compounds act on cell metabolism, affecting the cytoplasm [38], DNA and RNA [19], proteins, and carbohydrates of the extracellular matrix [13], and adherence to surfaces [40].

In recent years, special attention has been given to the search for novel strategies for the use of natural-based formulations for the control of caries-related variables. The anti-inflammatory and antimicrobial properties of some extracts have prompted the investigation of the incorporation of such compounds into mouthcare products such as mouthwashes and dentifrices. A recent clinical trial suggested that a dentifrice containing Red Brazilian Propolis did not interfere with the kinetics and bioavailability of the salivary F in samples, enabling its integration with the formulation without compromising its anticaries activity [44]. Another one found that a polyphenol-rich cranberry dentifrice influenced a species-level shift in the ecology of the oral microbiome, resulting in a microbial community less associated with dental caries [45]. In fact, a recent literature review has thoroughly summarized the main data on the use of organic toothpaste formulations, verifying a gap in the literature of clinical studies, which should be encouraged, in order to produce sufficient evidence to provide consumers with recommendations for daily use, based on both efficacy and biocompatibility [20].

In general, our data point to a significantly higher effect of the experimental toothpaste on the growth inhibition of most of the micro-organisms assessed. This supports the idea of the simultaneous administration of polyols, polyphosphate, and F at a reduced concentration (200 ppm F) as an alternative for managing cariogenic biofilms, especially for young children, as it would reduce systemic fluoride exposure, while sustaining its mineralizing effects (from F and TMP) and enhancing the antimicrobial effects (from all the actives) compared with a conventional (1100 ppm F) toothpaste. However, any extrapolations to clinical conditions would be extremely premature given the preliminary nature of the data obtained, and limitations inherent to the study protocol. Future studies with biofilms, especially formed on dental substrates, could bring important data in this regard.

## 4. Materials and Methods

The toothpastes were produced by Xlear Incorporation, American Fork, UT, USA, and contained in their composition the following actives: xylitol (X: 16%), erythritol (E: 4%), sodium trimetaphosphate (TMP: 0.25%), and sodium fluoride at 200 ppm F, alone or in different combinations. Formulations without any actives (placebo) or containing 1100 ppm F were also manufactured as controls. The groups were: (a) placebo (without any active compound; negative control toothpaste); (b) 1100 ppm F (positive control toothpaste); (c) 200 ppm F (200F); (d) TMP (0.25%); (e) 200F+TMP; (f) xylitol (16%); (g) erythritol (4%); (h) xylitol+erythritol; (i) 200F+xylitol+erythritol; (j) TMP+xylitol+erythritol; (k) 200F+TMP+xylitol+erythritol (experimental toothpaste).

### 4.1. Preparation of Toothpaste Slurries

Toothpaste slurries were produced by dispersing 10 g of each toothpaste in 10 mL of sterile deionized water (1:1). Serial dilutions of the slurry were made using sterile deionized water, to obtain four additional dilutions at 1:2, 1:4, 1:8, and 1:16 [46].

### 4.2. Antimicrobial Assay

Agar diffusion assays were performed with the reference strains of the American Type Culture Collection (ATCC): *C. albicans* (ATCC 10231), *S. mutans* (ATCC 25175), *A. israelii* (ATCC 10048), and *L. casei* (ATCC 393) [17]. These were reactivated from their original cultures in Agar Sabouraud Dextrose (ASD; Difco, Le Pont de Claix, France) for *C. albicans*, and in Brain and Heart Infusion Agar (BHI Agar; Difco) for *S. mutans*, *A. israelli*, and *L. casei*, and incubated in 5% CO_2_ at 37 °C, for 24 h. Following this, five colonies of each species were individually added to BHI broth and incubated at 37 °C for 18–24 h. Aliquots of 300 μL of each bacterial suspension (optical density of 0.6, and absorbance of 550 nm -10^7^ CFU/mL for *C. albicans,* and 10^8^ CFU/mL for *S. mutans*, *A. israelii,* and *L. casei*) was homogenized with 15 mL of BHI Agar at 45 °C. Subsequently, equidistant wells (*n* = 6) were made on agar using sterilized cylinders (4 mm in diameter) and filled with 80 µL of the slurries of each toothpaste, at dilutions of 1:1, 1:2, 1:4, 1:8, and 1:16 [46]. The plates were kept for 2 h at room temperature to allow the solutions to diffuse, and then incubated at 37 °C for 24 h. The experiments were performed in triplicate. For each inhibition halo, three independent measures were performed with the aid of a digital caliper (accuracy 0.01 mm) (MitutoyoCD-15B) [46].

### 4.3. Statistical Analysis

Data were analyzed using the STATISTICA software (version 8.0). Data were submitted to normality (Shapiro–Wilk) and homogeneity (Barlett) tests. The *p* values, respectively, for normality and homogeneity tests for each micro-organism were: *S. mutans* (*p* = 0.301; *p* = 0.970), *C. albicans* (*p* = *0*.296; *p* = 0.816), *L*. *casei* (*p* < 0.05; *p* = 0.220), and *A. israelii* (*p* = 0.658; *p* = 0.990). Data were subjected to two-way Analysis of Variance (ANOVA), considering as variation factors toothpastes and dilutions, followed by the Tukey’s HSD test, adopting a significance level of 5%.

## 5. Conclusions

The association of TMP, F, xylitol, and erythritol was shown to be effective in reducing the growth of isolated cariogenic micro-organisms under most of the conditions studied (i.e., micro-organisms and dilutions), being, therefore, a promising alternative for controlling biofilms related to dental caries. Despite the promising trends found in this work, these results should be interpreted with caution given the study limitations due to the nature of the present protocol. In this sense, further assessments including a wider microbial variety and a combined biofilm evaluation with a substrate such as dental enamel should be performed in order to confirm the present trends.

## Figures and Tables

**Table 1:** Zone of inhibition of toothpastes containing different active compounds, at 5 dilutions, on *Streptococcus mutans*.

Toothpaste Dilutions (in Deionized Water)					
Groups	D1	D2	D4	D8	D16
**PLA**	12.7 ± 0.3 ^Aa^ (11.8–13.6)	10.4 ± 0.4 ^Bab^ (9.4–11.5)	10.5 ± 0.8 ^Ba^ (8.7–12.4)	10.5 ± 0.5 ^Bab^ (9.3–11.6)	10.3 ± 0.5 ^Bab^ (9.1–11.6)
**X**	10.8 ± 0.6 ^Ab^ (9.2–12.3)	10.4 ± 0.5 ^Aab^ (9.0–11.7)	10.2 ± 0.4 ^Aa^ (9.2–11.2)	10.1 ± 0.3 ^Ab^ (9.4–10.8)	10.0 ± 0.3 ^Aab^ (9.5–10.7)
**E**	10.2 ± 0.2 ^Ab^ (9.8–10.6)	10.5 ± 0.5 ^Aab^ (9.2–11.6)	10.3 ± 0.4 ^Aa^ (9.1–11.4)	10.5 ± 0.5 ^Aab^ (9.4–11.5)	9.8 ± 0.2 ^Ab^ (9.3–10.1)
**TMP**	10.8 ± 0.2 ^Ab^ (10.3–11.1)	10.5 ± 0.5 ^Bab^ (9.3–11.7)	10.3 ± 0.4 ^Ba^ (8.6–11.9)	10.6 ± 0.4 ^Bab^ (9.4–11.7)	10.4 ± 0.3 ^Bab^ (9.4–11.0)
**200F**	10.2 ± 0.6 ^Ab^ (12.2–11.5)	12.2 ± 0.6 ^Bb^ (11.5–12.6)	11.7 ± 0.1 ^BCb^ (11.7–11.8)	11.7 ± 0.4 ^BCc^ (11.6–12.0)	11.9 ± 0.4 ^Cc^ (11.8–14.1)
**1100F**	11.1 ± 0.4 ^Ab^ (10.0–11.9)	11.2 ± 0.2 ^Aab^ (10.6–11.5)	11.1 ± 0.4 ^Aa^ (10.8–11.4)	11.6 ± 0.3 ^Aac^ (11.4–11.7)	11.3 ± 0.3 ^Aa^ (10.7–11.9)
**X+E**	11.4 ± 0.8 ^Aab^ (9.3–13.3)	10.2 ± 0.5 ^Aa^ (8.9–11.4)	11 ± 0.5 ^Aa^ (9.9–12.0)	10.5 ± 0.5 ^Aab^ (9.4–11.5)	10.2 ± 0.2 ^Aab^ (9.7–10.5)
**200F+TMP**	21.1 ± 0.2 ^Ac^ (20.9–21.2)	20.3 ± 0.4 ^Bc^ (19.2–21.5)	19.9 ± 0.3 ^Bc^ (19.7–20.0)	19.5 ± 0.5 ^Bd^ (19.4–19.9)	19.2 ± 0.2 ^Bd^ (19.1–19.4)
**200F+X+E**	11.4 ± 0.4 ^Aab^ (10.3–12.4)	11.4 ± 0.3 ^Ab^ (11.2–12.6)	11.3 ± 0.4 ^Cb^ (11.1–11.5)	11.3 ± 0.3 ^Cc^ (11.0–11.4)	11.2 ± 0.3 ^Cc^ (11.1–11.3)
**TMP+X+E**	11.1 ± 0.6 ^Ab^ (9.5–12.6)	11.0 ± 0.5 ^Aab^ (9.8–12.1)	10.7 ± 0.5 ^Aa^ (9.5–11.8)	10.6 ± 0.5 ^Aab^ (9.4–11.7)	10.4 ± 0.6 ^Aab^ (8.8–12.0)
**EXP**	10.7 ± 0.4 ^Ab^ (9.3–12.0)	10.2 ± 0.2 ^Aa^ (9.3–11.0)	10.2 ± 0.6 ^Aa^ (9.9–11.1)	10.1 ± 0.4 ^Aab^ (10.0–10.4)	10.0 ± 0.5 ^Ab^ (10.0–10.5)

Means ± standard deviations of the means (95% Confidence Intervals). Distinct capital letters indicate statistical differences among dilutions within each group. Distinct lower-case letters indicate statistical differences among each study group within each dilution (two-way ANOVA and Tukey’s HSD test, *p* < 0.05; *n* = 9). PLA: Placebo, X: Xylitol (16%), E: Erythritol (4%), TMP: Sodium Trimetaphosphate (0.25%), 200F: 200 ppm Fluoride, 1100F: 1100 ppm Fluoride, X+E: Xylitol (16%) + Erythritol (4%), 200F+TMP: 200 ppm Fluoride + Sodium Trimetaphosphate (0.25%), 200F+X+E: 200 ppm Fluoride + Xylitol (16%) + Erythritol (4%), TMP+X+E: Sodium Trimetaphosphate (0.25%) + Xylitol (16%) + Erythritol (4%), EXP: 200 ppm of Fluoride + Xylitol (16%) + Erythritol (4%) + Sodium Trimetaphosphate (0.25%).

**Table 2:** Zone of inhibition of toothpastes containing different active compounds, at 5 dilutions, on *Lactobacillus casei*.

Toothpaste Dilutions (in Deionized Water)					
Groups	D1	D2	D4	D8	D16
**PLA**	13.4 ± 0.3 ^Aab^ (12.6–14.2)	12.0 ± 0.1 ^Ba^ (11.8–12.1)	12.0 ± 0.4 ^Bab^ (10.8–13.4)	11.2 ± 0.2 ^Bab^ (10.5–11.8)	6.5 ± 0.4 ^Ca^ (5.4–7.4)
**X**	15.4 ± 0.2 ^Ad^ (14.7–16.0)	12.9 ± 0.1 ^Bab^ (12.6–13.1)	12.0 ± 0.4 ^Babc^ (11.1–13.0)	11.9 ± 0.2 ^Bb^ (11.5–12.1)	6.5 ± 0.2 ^Ca^ (5.9–7.1)
**E**	13.1 ± 0.3 ^Aab^ (12.9–13.2)	13.4 ± 0.4 ^Ab^ (13.4–13.8)	11.4 ± 0.4 ^Ba^ (10.2–12.5)	11.6 ± 0.3 ^Bab^ (11.4–11.7)	11.8 ± 0.3 ^Bb^ (11.5–11.8)
**TMP**	12.8 ± 0.3 ^Abc^ (12.6–12.9)	12.6 ± 0.1 ^ABab^ (12.4–12.7)	13.2 ± 0.2 ^Ac^ (13.0–13.3)	11.5 ± 0.9 ^Bab^ (9.2–13.6)	6.5 ± 0.3 ^Ca^ (5.8–7.1)
**200F**	12.0 ± 0.1 ^Ac^ (11.5–12.3)	12.3 ± 0.5 ^Aab^ (11.1–13.5)	11.5 ± 0.5 ^Aa^ (11.0–11.6)	6.7 ± 0.2 ^Bc^ (6.2–7.1)	6.8 ± 0.2 ^Ba^ (6.3–7.1)
**1100F**	13.9 ± 0.2 ^Aa^ (13.4–14.4)	12.9 ± 0.2 ^ABab^ (12.7–13.0)	11.9 ± 0.6 ^Ba^ (11.0–12.7)	10.7 ± 0.8 ^Ca^ (8.8–12.6)	6.8 ± 0.2 ^Da^ (6.6–6.9)
**X+E**	13.2 ± 0.1 ^Aab^ (13.0–13.3)	13.2 ± 0.1 ^Ab^ (13.0–13.3)	11.9 ± 0.1 ^Ba^ (11.6–12.2)	10.8 ± 0.2 ^Bab^ (10.2–11.4)	6.1 ± 0.7 ^Ca^ (4.4–7.7)
**200F+TMP**	11.9 ± 0.1 ^Ac^ (11.8–12.1)	12.1 ± 0.2 ^Aa^ (11.9–12.2)	11.9 ± 0.3 ^Aa^ (11.0–12.6)	11.0 ± 0.2 ^ABab^ (10.8–11.1)	9.9 ± 0.2 ^Bc^ (9.7–10.0)
**200F+X+E**	13.9 ± 0.2 ^Aab^ (13.4–14.4)	13.4 ± 0.4 ^Bc^ (13.1–13.6)	13.3 ± 0.4 ^Ac^ (13.2–13.5)	11.5 ± 0.1 ^Cab^ (11.3–11.6)	6.2 ± 0.3 ^Da^ (5.2–7.1)
**TMP+X+E**	13.1 ± 0.2 ^Aab^ (12.9–13.2)	14.8 ± 0.3 ^Bd^ (14.5–14.8)	12.4 ± 0.9 ^Aabc^ (10.2–14.5)	12.2 ± 1.0 ^Bd^ (12.6–17.7)	6.5 ± 0.3 ^Ca^ (5.9–7.1)
**EXP**	14.2 ± 0.2 ^Ad^ (14.0–14.4)	13.5 ± 0.3 ^Bab^ (13.1–13.9)	13.1 ± 0.3 ^Bbc^ (13.0–13.2)	11.0 ± 0.2 ^Cab^ (10.8–11.1)	9.3 ± 0.4 ^Dc^ (9.1–9.5)

Means ± standard deviations of the means (95% Confidence Intervals). Distinct capital letters indicate statistical differences among dilutions within each group. Distinct lower-case letters indicate statistical differences among each study group within each dilution (two-way ANOVA and Tukey’s HSD test, *p* < 0.05; *n* = 9). PLA: Placebo, X: Xylitol (16%), E: Erythritol (4%), TMP: Sodium Trimetaphosphate (0.25%), 200F: 200 ppm Fluoride, 1100F: 1100 ppm Fluoride, X+E: Xylitol (16%) + Erythritol (4%), 200F+TMP: 200 ppm Fluoride + Sodium Trimetaphosphate (0.25%), 200F+X+E: 200 ppm Fluoride + Xylitol (16%) + Erythritol (4%), TMP+X+E: Sodium Trimetaphosphate (0.25%) + Xylitol (16%) + Erythritol (4%), EXP: 200 ppm of Fluoride + Xylitol (16%) + Erythritol (4%) + Sodium Trimetaphosphate (0.25%).

**Table 3:** Zone of inhibition of toothpastes containing different active compounds, at 5 dilutions, on *Actinomyces israelii*.

Toothpaste Dilutions (in Deionized Water)					
Groups	D1	D2	D4	D8	D16
**PLA**	5.9 ± 0.1 ^Aa^ (5.8–6.1)	5.6 ± 0.2 ^Aa^ (5.3–6.0)	5.8 ± 0.3 ^Aa^ (5.2–6.5)	6.1 ± 0.2 ^Aa^ (5.6–6.4)	6.1 ± 0.1 ^Aa^ (5.8–6.4)
**X**	5.9 ± 0.2 ^Aa^ (5.5–6.3)	5.9 ± 0.1 ^Aa^ (5.7–6.1)	6.1 ± 0.2 ^Aa^ (5.7–6.5)	5.9 ± 0.1 ^Aa^ (5.6–6.2)	6.0 ± 0.3 ^Aab^ (5.1–6.9)
**E**	6.2 ± 0.1 ^Aa^ (6.2–6.2)	6.1 ± 0.2 ^Aa^ (5.7–6.4)	6.0 ± 0.2 ^Aa^ (5.7–6.4)	5.9 ± 0.1 ^Aa^ (5.6–6.2)	6.0 ± 0.1 ^Aab^ (5.5–6.3)
**TMP**	5.9 ± 0.2 ^Aa^ (5.8–6.3)	6.0 ± 0.4 ^Aa^ (5.6–6.4)	5.9 ± 0.3 ^Aa^ (5.7–6.0)	6.0 ± 0.2 ^Aa^ (5.4–6.6)	5.9 ± 0.3 ^Aab^ (5.6–6.1)
**200F**	6.2 ± 0.1 ^Aa^ (6.0–6.3)	5.8 ± 0.1 ^Aa^ (5.7–6.0)	5.7 ± 0.1 ^Aa^ (5.2–6.1)	6.7 ± 0.2 ^Bb^ (6.2–7.1)	6.7 ± 0.4 ^Bc^ (6.2–6.9)
**1100F**	5.7 ± 0.3 ^Aa^ (5.5–6.0)	6.0 ± 0.3 ^Aa^ (5.8–6.3)	5.8 ± 0.2 ^Aa^ (5.4–6.2)	5.9 ± 0.2 ^Aa^ (5.3–6.6)	6.0 ± 0.2 ^Aab^ (6.0–6.2)
**X+E**	6.0 ± 0.1 ^Aa^ (5.8–6.1)	5.7 ± 0.2 ^Aa^ (5.2–6.2)	5.8 ± 0.4 ^Aa^ (5.6–6.1)	5.7 ± 0.3 ^Aa^ (5.4–6.0)	5.9 ± 0.2 ^Aab^ (5.5–6.4)
**200F+TMP**	5.7 ± 0.2 ^Aa^ (5.2–6.3)	5.7 ± 0.2 ^Aa^ (5.2–6.2)	5.6 ± 0.2 ^Aa^ (5.2–6.1)	5.8 ± 0.3 ^Aa^ (5.3–6.3)	5.5 ± 0.4 ^Ab^ (5.1–6.0)
**200F+X+E**	5.7 ± 0.2 ^Aa^ (5.3–6.2)	5.8 ± 0.3 ^Aa^ (5.0–6.6)	5.7 ± 0.3 ^Aa^ (5.5–6.0)	5.9 ± 0.1 ^Aa^ (5.7–6.0)	5.1 ± 0.2 ^Aab^ (5.6–6.1)
**TMP+X+E**	6.0 ± 0.2 ^Aa^ (5.8–6.1)	5.9 ± 0.1 ^Aa^ (5.7–6.2)	5.8 ± 0.3 ^Aa^ (5.6–6.0)	5.9 ± 0.3 ^Aa^ (5.7–6.0)	5.9 ± 0.3 ^Aab^ (5.7–6.0)
**EXP**	5.8 ± 0.1 ^Aa^ (5.6–5.9)	6.0 ± 0.3 ^Aa^ (5.6–6.4)	5.9 ± 0.1 ^Aa^ (5.8–6.1)	5.9 ± 0.3 ^Aa^ (5.5–6.3)	6.1 ± 0.3 ^Aa^ (5.8–6.4)

Means ± standard deviations of the means (95% Confidence Intervals). Distinct capital letters indicate statistical differences among dilutions within each group. Distinct lower-case letters indicate statistical differences among each study group within each dilution (two-way ANOVA and Tukey’s HSD test, *p* < 0.05; *n* = 9). PLA: Placebo, X: Xylitol (16%), E: Erythritol (4%), TMP: Sodium Trimetaphosphate (0.25%), 200F: 200 ppm Fluoride, 1100F: 1100 ppm Fluoride, X+E: Xylitol (16%) + Erythritol (4%), 200F+TMP: 200 ppm Fluoride + Sodium Trimetaphosphate (0.25%), 200F+X+E: 200 ppm Fluoride + Xylitol (16%) + Erythritol (4%), TMP+X+E: Sodium Trimetaphosphate (0.25%) + Xylitol (16%) + Erythritol (4%), EXP: 200 ppm of Fluoride + Xylitol (16%) + Erythritol (4%) + Sodium Trimetaphosphate (0.25%).

**Table 4:** Zone of inhibition of toothpastes containing different active compounds, at 5 dilutions, on *Candida albicans*.

Toothpaste Dilutions (in Deionized Water)					
Groups	D1	D2	D4	D8	D16
**PLA**	5.6 ± 0.2 ^Aa^ (4.9–6.4)	5.5 ± 0.1 ^Aa^ (5.1–5.9)	5.6 ± 0.3 ^Aa^ (5.3–5.8)	5.8 ± 0.7 ^Aa^ (5.7–6.0)	5.6 ± 0.3 ^Aab^ (5.5–5.8)
**X**	5.7 ± 0.1 ^Aa^ (5.6–5.9)	5.7 ± 0.3 ^Aa^ (5.3–6.2)	5.7 ± 0.3 ^Aa^ (4.9–6.4)	5.9 ± 0.5 ^Aa^ (5.7–6.8)	5.4 ± 0.4 ^Aa^ (5.2–5.5)
**E**	5.9 ± 0.3 ^Aa^ (5.2–6.5)	5.8 ± 0.3 ^Aa^ (5.4–6.2)	5.9 ± 0.5 ^Aab^ (5.2–6.7)	5.8 ± 0.3 ^Aa^ (5.0–6.6)	5.9 ± 0.3 ^Aab^ (5.2–6.7)
**TMP**	6.2 ± 0.2 ^Aab^ (5.5–6.9)	5.1 ± 0.1 ^ABa^ (5.1–6.0)	5.1 ± 0.2 ^ABab^ (5.0–6.1)	5.4 ± 0.2 ^Ba^ (5.2–6.5)	5.7 ± 0.5 ^ABab^ (5.3–6.1)
**200F**	5.8 ± 0.1 ^Aa^ (5.5–6.1)	5.5 ± 0.1 ^Aa^ (5.1–5.9)	5.6 ± 0.9 ^Aa^ (5.3–5.9)	5.8 ± 0.1 ^Aa^ (5.4–6.2)	5.6 ± 0.7 ^Aab^ (5.4–5.7)
**1100F**	6.1 ± 0.2 ^Aab^ (5.5–6.7)	5.9 ± 0.2 ^Aab^ (5.7–6.2)	6.1 ± 0.3 ^Aab^ (5.4–6.8)	5.8 ± 0.2 ^Aa^ (5.4–6.3)	5.8 ± 0.1 ^Aab^ (5.5–6.0)
**X+E**	6.0 ± 0.5 ^Aab^ (5.8–6.3)	5.1 ± 0.3 ^Aa^ (5.1–6.0)	5.1 ± 0.2 ^Aa^ (5.1–5.4)	5.1 ± 0.3 ^Aa^ (5.0–5.7)	5.0 ± 0.9 ^Aab^ (5.0–5.4)
**200F+TMP**	5.8 ± 0.1 ^Aa^ (5.5–6.1)	5.5 ± 0.4 ^Aa^ (5.1–5.9)	5.6 ± 0.2 ^Aa^ (5.3–5.9)	5.8 ± 0.1 ^Aa^ (5.4–6.2)	5.6 ± 0.7 ^Aab^ (5.4–5.7)
**200F+X+E**	6.6 ± 0.1 ^ABb^ (6.4–6.7)	6.6 ± 0.2 ^Bb^ (6.3–6.9)	5.9 ± 0.4 ^ACab^ (5.6–6.2)	5.7 ± 0.3 ^Ca^ (5.3–6.1)	5.9 ± 0.2 ^ACab^ (5.3–6.5)
**TMP+X+E**	5.7 ± 0.2 ^Aa^ (5.4–5.9)	5.7 ± 0.2 ^Aa^ (5.2–6.2)	5.8 ± 0.2 ^Aa^ (5.5–6.0)	5.8 ± 0.1 ^Aa^ (5.5–6.5)	5.7 ± 0.7 ^Aab^ (5.2–6.2)
**EXP**	7.9 ± 0.4 ^Ac^ (6.7–9.1)	7.6 ± 0.3 ^Ac^ (6.6–8.5)	6.6 ± 0.2 ^Bb^ (6.1–7.1)	6.0 ± 0.7 ^Ba^ (5.4–6.7)	6.0 ± 0.1 ^Bb^ (5.9–6.5)

Means ± standard deviations of the means (95% Confidence Intervals). Distinct capital letters indicate statistical differences among dilutions within each group. Distinct lower-case letters indicate statistical differences among the study groups within each dilution (two-way ANOVA and Tukey’s HSD test, *p* < 0.05; *n* = 9). PLA: Placebo, X: Xylitol (16%), E: Erythritol (4%), TMP: Sodium Trimetaphosphate (0.25%), 200F: 200 ppm Fluoride, 1100F: 1100 ppm Fluoride, X+E: Xylitol (16%) + Erythritol (4%), 200F+TMP: 200 ppm Fluoride + Sodium Trimetaphosphate (0.25%), 200F+X+E: 200 ppm Fluoride + Xylitol (16%) + Erythritol (4%), TMP+X+E: Sodium Trimetaphosphate (0.25%) + Xylitol (16%) + Erythritol (4%), EXP: 200 ppm of Fluoride + Xylitol (16%) + Erythritol (4%) + Sodium Trimetaphosphate (0.25%).

## Data Availability

The data presented in this study are available upon request from the corresponding author.
